# PPI in psychiatry and the problem of knowledge

**DOI:** 10.1186/s12888-023-05398-0

**Published:** 2024-01-15

**Authors:** Diana Rose, Peter Beresford

**Affiliations:** 1 CASS, ANU, ACT, Canberra, 0200 Australia; 2 University of East Anglia, Research Park Norwich, Norwich, NR4 7TJ UK

**Keywords:** Psychiatric survivors, Knowledge, Involvement

## Abstract

This article begins by locating Patient and Public involvement ((PPI) historically and argues that ‘mental health’ was a special case. This movement held promise for service users in repositioning them as researchers as opposed to ‘subjects’. We argue, however, that ultimately it failed and was reduced to involved publics ‘tinkering at the edges’. In respect to this we reference institutions, hierarchies, organisations and the overall political climate. Ultimately, however, it failed at the level of knowledge itself in that t he underlying assumptions of conventional researchers, their aims and goals, clashed with those of the assumptions and aims of survivors. However, we argue that all is not lost, the mainstream itself is imploding and beneath the surface forms of distinctly survivor-led knowledge are emerging.

## Introduction

The title of this special issue brings to mind, probably deliberately, what has come to be known as PPI in research. That is, Patient and Public Involvement in research, here specifically psychiatric research. This activity had a along gestation. It can be dated from 1986 when the UK Department of Health established the Advisory Body ‘Consumers in NHS Research’ but it became fully institutionalised in 1996 with the formation of NIHR, the National Institute for Health Research, which was responsible for funding all research in the UK National Health Service, and its ‘PPI’ programme called INVOLVE. We do not use the word ‘institution’ lightly as will become clear. This slow emergence represented, for some, a space of promise. Research was no longer to be done ‘on’ subjects but patients and the public were to be given the status of researchers themselves. And new methods were to be explored, although this was not central. Critical was that the Department poured a lot of money into this endeavour and people bidding for research funds were obliged to show how they would involve patients and the public in their work as an ostensible condition of receiving an award.

To clarify, by the mainstream we mean the dominant unreconstituted psych narrative which underpins both prevailing Global North mental health ‘treatment’ systems as well as colonized Global South ones; ruling typologies of disorder and research approaches. It extends through political and policy structures, through systems of ‘expert’ publication and peer review, to related scholars, clinicians and learners, perpetuating a powerful nexus of attitudes, assumptions and ways of working. It does not always manifest in this ‘strong’ form but analysis reveals that superficially ‘patient-centred’ research and practice is based on the same underpinning assumptions. Its counter, including survivor activists and researchers, self-help schemes and nascent attempts at co-production, is to be found in new social movements of survivors and disabled people, their under-funded user-led organisations and people like us working for a foothold to challenge mainstream orthodoxy, from within as well as without its structures.

### Myth or reality?

All of this was portrayed as something new, a ‘first time’ event. And as an institution it was new, but as a vision it is more of an origin myth. Patients and the public had been involving themselves in research long before this, in and outside mental health. Both the current authors were carrying out, or writing about, such involvement in mental health research during the late 1980s and early 1990s [1, 2]. Arguments for the demedicalisation of childbirth started with the Boston collective in 1973. Empirical work was slow to take off but when it did it was not standard. An intensive interview study of experiences of childbirth was published in 2004 [3]. It is important that this was an interview study as it breached the canons of ‘hard science’ and it had a political base. In fact, the authors drew on an important body of feminist theoretical work which began in the early 1980s.

Sandra Harding published an early collection called *Feminism and Methdology*, discussing whether aspects of methodology would need to change for work to be definitively feminist [4]. But her main interest became knowledge and it is quite illuminating that feminists chose to address their oppression at the level of knowledge as such [5, 6] Harding and subsequent writers proposed a ‘feminist standpoint epistemology’ which meant that the political position of feminism produced a different kind of knowledge to that of the mainstream. Herstory not history. It is important to say that this was a political position because, unlike some, these writers did not believe that a different knowledge was intrinsic or essential to women in some way. It was directly linked to activism, discussion and reflection such that knowledge emerged in the course of these activities, or to use Harding’s own word was *achieved* [7].This was controversial, of course, and has been discussed latterly in respect of mental health by Jijian Voronka [8]. But the general idea that a group’s positionality shapes their knowledge about themselves and more widely has been influential. It does, of course, propose the opposite of mainstream emphases on objectivity, neutrality and generalisability [9, 10]

When it comes to the domain of welfare, the pioneer among the welfare user movements in seeking a different kind of relation with research, policy and practice, was the international disabled people’s movement. While, for example, the flowering of mental health survivors’ movements can be seen to have taken place during the 1980s, this had been anticipated by more than a decade by the disabled people’s movement. The fundamental principles of disablement were published in 1976 [11], but efforts first to use research in a campaigning way and then to develop their own research had been pioneered by disabled people even earlier in the late 1960s [12]

### Different developments

There was another perhaps even more significant difference between these two pioneering movements, taking the UK as an example. While the psychiatric system survivors/mental health service user movement was deeply embedded in the psychiatric system, the disabled people’s movement can be seen as much more self-consciously separatist. It developed its own social model of disability and philosophy of independent living; it rejected the approach of conventional and reformist policymakers and theoreticians and even developed a model of meeting disabled people’s rights and needs which stood outside existing professions, provision and service system: direct payments [13, 14].

These bodies of work, whether intentionally or otherwise, were effectively sidelined by PPI. It was partly a question of method and partly of forms of underpinning knowledges. We may also even suggest that there was only a limited awareness of such grassroots developments among researchers. To say ‘sidelining’ is putting it weakly because a formidable form of power was also being exercised. We must remind ourselves here of the massive inequalities of power operating between practitioners, researchers and service users/patients in this context. Certain methods and certain knowledge forms were erased. These are linked. We will argue that these examples show that PPI could have led to radical change but instead it actually remained largely within the constraints of the mainstream and so it did not transform anything at all. Indeed, institutionally it failed, its main programme closed and its chief activity was reduced to writing ‘plain English summaries’ of research. This happened only two years ago but it was predictable long before that [15].

However, the ‘underside’ did not go away. Under the surface, alternative forms of knowledge and knowledge production were growing and being discussed, in the face of many obstacles. They represent an epistemic collision with mainstream science. We will argue, then, that PPI failed, even in its own objectives, because it wanted to ‘stay safe’, to bury power and avoid collision at a time of increasingly polarised political ideology.

### Psychiatry specifically

In this configuration, mental health occupies a specific space. For some this is ultimately because of the power of psychiatry to detain and ‘treat’ against a person’s will [16]. But power operates at many levels as we shall see. Two things are important. One is that there has always been controversy between psychiatry and its recipients. At one level this is because of what the ‘aim’ is supposed to be. In much mainstream medicine, there is agreement on ‘outcomes’ between doctor and patient. Most obvious are survival and quality of life (yes, these can be in conflict). But there is no such agreement between politicised survivors and medical psychiatrists. We want different things – minimizing side-effects, attention to our material lives, the right to be angry and not to be zombies. At the very least we certainly want more than being diagnosed. Iatrogenic medicine is alive and well in psychiatry and bears a deal of responsibility for ‘chronicity’.

Second, though, survivors who enter research are entering the very space where they are supposed to be incompetent, that is, of knowledge-making itself. Positioned as unable to think, lacking insight, cognitively deficient we seem to have the *chutzpah* to believe we can do as well as the scientists. *They* are the epitome of rationality: something we definitively lack. So epistemology plays a variety of roles in this argument [17].

By the end of this paper it should be clear that mainstream research occupies a different epistemic space to that of survivor research (by which we include conceptual work) and these spaces clash. They are founded on different premises. Mainstream research thinks it has no premises – it is objective and value-free – but they are there, just invisible. To be blunt, mainstream research is lacking in the face of concepts. It can’t handle them so just churns out more and more empiricist work endorsed by peer review at the level of both funding and publications [18, 19].

Before moving on, it could be asked if this failure is specific to psychiatry and associated forms of knowledge such that other ‘disciplines’ – parts of social science and the humanities – have been more successful in “letting madness speak and direct”. This is complex and we are not polymaths. However, first, the *quantity* of publications in the biomedical sphere on PPI far outstrips that in other disciplines [20]. What about the quality, reach and transformative potential? Probably the biggest project in the Humanities in this sphere was the Wellcome-funded “Hearing the Voice” led by Angela Woods at Durham [21]. It aimed to shine a new lens on hearing voices. There was an Advisory Group which contained a few voice-hearers and one of the lead researchers also had this experience. The Advisory Group, in addition, contained many of the ‘big names’ in the field. For our purposes, this project was not particularly transformative. It focussed heavily on stigma and the voice-hearing researcher put huge energy into setting up support and anti-stigma groups for voice-hearers based in the university sector. It was run from Oxford. There is nothing at all wrong with this in its own terms but it hardly meets goals of inclusivity and equality which are issues that remained unresolved in biomedical PPI too.

The inter-disciplinary literature now has a considerable literature on ‘narrative’, where a favourite topic is the ‘illness narrative’ and relatedly so-called ‘recovery narratives’ are receiving attention. Llewelyn-Beardsley and colleagues conducted a systematic review of the characteristics of a ‘recovery narrative’. In 2019 [22]. The review is complex and interesting and much could be said. For our purposes, though, the PPI consisted of a single Lived Experience Advisory Group which was purely consultative. For example, it had no role in the analysis of the selected papers. Further, it is completely taken for granted that ‘recovery is a good thing’. They invoke Mad Studies but not the argument from that body of work which holds that recovery stories are so prescribed that they almost amount to ‘disability porn’ [23]. The entire literature on what the activist group Recovery in the Bin call the “Unrecovered” and why some people cannot measure up is ignored. Finally, although the review contests the ‘medical model’ it is not averse at all to a ‘*clinical*’ model, advocating various forms of ‘narrative therapy’. Again, the community-development literature, seemingly promising here, has been critiqued by Rachel Aldred for its basis in social psychology and ‘positive psychology’. In other words, back to ‘psy’ – old wine in new bottles [24].

If these examples are representative, then the hope that the grass is greener in the Humanities side of the fence seems not fulfilled at the moment. A different case is that of law and particularly the study of human rights. This changes the positionality of the person from patient in medicine to bearer of rights. This is complex as ‘protective rights’ can include detention and involuntary treatment. However, there have been progressive moves to accord positive rights to people with disabilities, including ‘psychosocial disabilities’ [25, 26]. This culminated in the publication in 2008 of the UN Convention on the Rights of People with Disabilities. The drafting process itself could be called ‘participatory’ as most Committee Members were people with disabilities and / or representatives of disabled people’s organisations (Gooding, op.cit.). The Convention is the first time that disabled people are accorded equality with everyone else in international law. *If* implemented, this would indeed be a paradigm shift but implementation is a central problem. It is a very controversial document and we cannot do it justice here but will return to this treaty in discussions of differences of emphases regarding the CRPD between some in the Global South and some in the Global North below (acknowledging that this distinction is unsatisfactory).

### The challenge facing the survivors’ movement

To return to the disabled peoples’ and survivor movements in relation to PPI in research, it is not difficult to see why these two (overlapping) movements actually took different directions generally and then more specifically in relation to participation. First, many mental health service users were dependent on the psychiatric system, in a different way to other disabled people. Indeed, those under section or compulsorily restrained in some other way, could not necessarily even leave it. Second, they already had a history of developing working relations with some progressive practitioners and professional pioneers and their campaigning efforts for change had involved and been supported by them. Third, the psychiatric system offered a route to resources otherwise unavailable – it developed budgets for involvement and engagement, much of it funded by government. Also, psychiatric hospitals could control access to service users, so to safeguard the rights of peer in-patients and keep in contact with them, survivors and their organisations had to build working relationships with the service system [27].

So, from the start it could be argued that opportunities for independent action, resourcing and philosophy were at risk of being compromised. Certainly the survivor movement did not have and had been cautious about developing its own distinct philosophy along the lines initiated by the international disabled people’s movement. The survivor movement also faced a more subtle barrier limiting its freedom of action. While people with physical and sensory impairments might be seen as having defective or ‘broken’ bodies, mental health service users/survivors could expect to be seen and treated as having impaired reason and defective minds. This helps explain their often ambiguous relationship with professionals and professional power. It may also explain why explicitly radical survivor organisations like Survivors Speak Out had relatively short lives and instead pressure for involvement was coopted into traditional mainstream research and associated state and voluntary services which were more like the traditional, large disability charities, that the disabled people’s movement despised and rejected in favour of independent ‘user-led’ organisations of their own, clearly aligned with their movement.

### Ideological conflicts

All this it should be remembered was happening at the same time as in the UK and internationally neoliberalism with its commitment to small state politics, privatization, market-led services, cuts in public services and expenditure was in the ascendancy. And it is perhaps this overarching reality which helps explain why the promise of PPI both generally and specifically in research comes to be called into question. The idea of PPI of involving the perspectives of those on the receiving end of policy, practice and knowledge formation in the process, can play well with the consumerist rhetoric of neoliberal ideology. Here the argument is that the public policy consumer no less than the consumer of other goods and services needs to be listened to if service providers and funders are most effectively to match the demand. However, all the talk of the ‘customer is king’ (sic), market research and listening to the consumer, associated with such an understanding of involvement is something very different from the empowerment and equalization of power that motivated members of service user movements to get involved. They wanted more say in their lives and services and that is not the same as helping market organisations target their goods and services with maximum economy and efficiency.

### Linguistic confusion and power

It quickly became apparent to those that got involved in PPI that the same words were being used by them and the service system to mean very different things. As was said a long time ago the politics of the supermarket are not the same as the politics of liberation [28]. PPI might not offer what people as mental health service users/survivors wanted, but truth to tell, it never said it would. The promise of effective involvement failed here because the prevailing political ideology was neoliberal rather than emancipatory, but that is not to say any deception was deliberately played on participants. Of course, in this instance the potential for misunderstanding and the resulting problem was made much worse because of the massive imbalance of power between would-be participants and the psychiatric system and the key professionals operating it.

Within the psychiatric system, inequalities of power have long been pronounced. These are not only the result of traditionally hierarchical medical structures of organisation and management, but also followed from the priority given to some professions over others, notably psychiatry and the overlaying of the psychiatric system with the ‘new public management’ associated with neoliberalism, with its emphasis on micro-management, surveillance, control and extended bureaucracy [29]. ‘Mental health’ research has not escaped this paradigm and service users/patients have been at the bottom of all these hierarchies.

Perhaps we need to say an additional word here about language. As we and others in this field have long suggested, terminology in relation to identity is a battleground in this as in other identity-based political discussions. Many terms are attached to the subjects of ‘mental health’ provision, from the frankly abusive, through pathologizing medicalised labels, to the kind of language people have used themselves as part of seeking to reclaim who they are and how they/we might be understood. There is no consensus here, nationally or internationally, either in relation to what terms are used or how they are understood. It is almost inevitable that someone will feel uncomfortable or even insulted whatever the terms employed. From our experience in the NSM of ‘mental health service users’ our preference is to talk of service users/survivors. We don’t attach particularly different meanings to these two terms, although we know some involved do. Some will define themselves as psychiatric system survivors to indicate the oppression they experience as ‘service users’. Others dismiss service users as a passive term, confusing people with illicit drug use. We use the latter term in the way suggested by the user-led UK organization Shaping Our Lives, as a *political* term (https://shapingourlives.org.uk/about-us-and-inclusive-involvement/definitions-and-meanings/#:~:text=The%20term%20%27service%20user%27%20can,(or%20have%20used%20them).) and we often couple these two terms service user/survivor if only to indicate our desire to be inclusive and to acknowledge and respect other people’s preferences. Ultimately, we are wary of letting linguistic disagreements be used by professionals, as they sometimes are to delay and stifle the possibility of action and solidarity. On the other hand, the different terminologies do signify different positions through their connotations and political placements, history and geography. We have already shown this with the term ‘consumer’ and its links to the market but it was the preferred term in Australia and New Zealand for many years. A detailed analysis of this can be found in Chapter 6 of Rose [15]

### The strength of resistance

The fact that service users would be seeking a shift in power within services and generally this was not on offer, meant that over time there was an increasing tendency for them to feel that PPI arrangements were essentially tokenistic, ineffective and from their perspectives, unsuccessful. While it could be argued that this was the result of a misunderstanding about what nature of participation was on offer, a more fundamental issue also undermined the venture. This concerned whose knowledge such PPI was concerned with advancing. And here was where the gulf between these two philosophies for involvement became most clear. If service users/survivors were interested in developing their knowledge in order to advance their agendas and priorities, not surprisingly those involved in the psychiatric system were more concerned with advancing their professional and philosophical concerns [30, 31]. Having highlighted the ‘epistemic injustice’ that this reflects and results in, ironically, researchers now seem bent on taking over this domain of enquiry too [31].

Despite now a generation of interest in PPI and user involvement in psychiatric research having passed, the truth is that the psychiatric system is still dominated by a medicalised individual model; the psychiatric empire has if anything expanded, both in the global north and south and the research activities of the survivor movement and its user-controlled organisations largely remain marginal and insecure. This is in some contrast to the situation as regards physical and sensory impairments where there have been significant policy and practice changes in global north countries and some global south nations have challenged psy values and approaches both through their own indigenous understandings and under the umbrella of the UN Convention of the Rights of Disabled People as well as regional organisations such as Towards Inclusion Asia (TIA) [32]: https://tci-global.org/bali-declaration/). It should also be noted that there are some tensions or differences of emphases in respect of the CRPD between some in the ‘Global North’ and some in the ‘Global South’. For example, Article 12 and particularly the General Comment on this Article, accords equal capacity before the law to disabled people, including those with mental health problems. In the Global North, discussions here tend to be focused on ‘psychiatric emergencies’ and involuntary detention and treatment, variously arguing that such coercion should be abolished [33] or that it actually compromises the right to health [34], In the Global South the right to legal capacity has been discussed far more broadly and encompasses other Articles – in respect of signing contracts, marriage and the right to occupy a directive role in an organisation, for example. First, it is argued that there are far fewer psychiatric facilities in the Global South so ‘emergencies’ themselves are less prominent. Second, though, Article 19 – the right to community participation is more prominent in the Global South. It is not for nothing that TIA is called Towards *Inclusion* Asia (see also Davar, [35, 36]. This is brief, but points again to the role of ‘culture’ (including material factors such as poverty and homelessness) in the implementation of an International Treaty.

### The particular problem for survivors

While there has been a tendency historically to present disabled people, that is to say people with physical and sensory impairments as frightening, deviant and defective, in, for example, literature, film and the arts and to associate their perceived difference and deficiency in moral terms, from Long John Silver and the Hunch Back of Notre Dame to the Phantom of the Opera and Freddy Krueger, mental health service users can additionally expect to be positioned as irrational and dangerous. Herein lies the often unstated but looming objection to their full and equal inclusion in research and knowledge production. They are unable to think rationally and intelligently and yet are expecting to be part of research processes for knowledge production which have consistently highlighted their scientific, rigorous and reliable underpinnings. Mental health service users are in an impossible position given the scientific narrative. The ‘mad scientist’ maybe is a joke but she is certainly an oxymoron.

### The significance of method

Knowledge in mainstream thought is intimately linked with methodology, so we will now look in more detail at method. Firstly, this is held to guarantee neutrality. In these days of Evidence-Based Medicine, there exists a hierarchy – a hierarchy of methods. The Randomised Controlled Trial is trumped only by the Systematic Review, epidemiology has a prominent place and observational work a lower one. There is no mention at all of participatory research – indeed, qualitative research as a whole or in components does not appear. And this hierarchy and its exclusions is not simply an academic graphic [37]. It has a strong effect on funding so proposals using RCTs and cohort studies have a much stronger chance of being successful financially. It clashes with the kind of work done by survivors [38]

The literature discussed earlier was all to some degree ‘participatory’. Invisible in the Cochrane hierarchy, less likely to be funded. Where you *might* find it in the INVOLVE narrative is in the idea of ‘user-led research’. But this was relegated to the settings of NGOs. This looks odd at first but actually is a clever way of devaluing such knowledge and research. It is not just missing from the hierarchy, it does not belong in the academy, the epitome of knowledge production.

We make these remarks to demonstrate that PPI had little chance of changing the methods, either in themselves or with their consequences for funding, and so was reduced to a kind of tinkering at the edges [39]. It is not often thought that method is wrapped up with power and money but this was the case in the field we are looking at here.

### The limits of positivist research

One aspect of the Anglophone empiricist tradition requires special mention. Because of its commitment to objectivity, randomisation and freedom from bias (all of which may be questioned) it is claimed that the results of RCTs in particular are generalisable. Because randomisation is said to control for all ‘extraneous factors’, the suggestion is that what happens in a given trial will happen almost anywhere. This has particular purchase in respect of the influence of Western Psychiatry on the Global South. There are papers that claim that if x works in South London it will also work in South Sudan. This is referred to as ‘scaling up’; scaling up research from the West to practice in the South [40]. Predictably, there is much controversy here ranging from those who see this movement – for Global Mental Health – as bringing help to neglected and abandoned peoples to those who see it as frank colonisation. PPI has not had much traction in this space, but there are ‘peer networks’ from across the world that advise on global research programmes. Although not much is made public about how these operate, one paper by survivors at least suggests that what has been ‘scaled up’ in these endeavours is the power relation between psychiatry and its recipients, shot through with the usual tactic of silencing dissent [41]

It might also be noted that mad people and colonised people have something in common. Both are said to ‘lack reason’—something touched on already. Whether it be the ladder of civilisation or the confinement of the asylum, we are positioned as lacking the capacity to think. Of course, ordinary members of the public would vigorously deny that they think such things or that they influence their efforts in PPI. What we are talking about is systemic though and part of Eurocentric thought which is such an abstract idea most would give it no quarter. But we are underpinned by assumptions and things we take for granted. So the next section will compare the premises of psychiatry with the premises of Mad Knowledges to bring the differences into relief and add to our explanation of why PPI has so far failed.

### Conflicting assumptions

Psychiatry is replete with assumptions. Some are articulated at length – like the DSM. But the **assumption** is that mental illnesses are categorical entities, pathological ones. And they reside in individuals who are also the target of treatments. The distinction between normal and pathological is fundamental. Not very complicated, but as with all assumptions they have the status of the obvious to those who abide by them. However, this paper is about patient involvement in research so what about the taken-for-granted in research? They are equally not very complicated. First, is that knowledge will accrue by the performing of experiments. We will ‘test’ the world, reject or accept a number of Null Hypotheses and reality will reveal itself. Thus is the scientist neutral and objective because their role is to let the real show itself. Certain safeguards are needed to avoid bias, randomisation and ‘blinding’ being key. The unit of analysis is the individual and in this framing they are passive. We put this briefly because critiques of the ‘medical model’ are well known. But it is important to draw the distinction between psychiatry and psychiatric research because it is the latter that PPI was designed to alter. Of course they are linked, but research could proceed as a series of case studies and once did. That the assumptions take the form they do is a matter of epistemology.

Survivor research like emancipatory disability research before it, was premised on some key principles. But they were not the principles of the mainstream. From survivors’ perspective, it would be concerned with undertaking research in different ways for different purposes. Thus, the aim was to equalize relations between researcher and researched and to undertake research that was personally empowering in its process and aims and committed to political change and the political empowerment of those involved and their broader constituency [42]. Key to this of course was the concern to liberate and advance survivors’ own knowledges, rather than as historically psych system research has mainly done, to advance the interests and concerns of the psychiatric system and profession and the related concerns of big pharma.

This is why historically the latter research has focused particularly on addressing the medical model preoccupations of the two big systems – psych and pharma – and advancing their knowledge, rather than telling us more about what survivors want and think. Insofar as this has been addressed by prevailing research it has been through professionals seeking their ‘views’ through their methods and methodologies and analysing and interpreting these themselves [43]. The inherent problems of bias in such an approach are readily apparent, although long overlooked through acceptance of the assumption of the authority of traditional positivist research. Thus, researchers associated with the psych system tended to reinforce its belief system and legitimize and give authority to their own assumptions.

Working from their own lived experience, the emergence of the survivor movement served as an opportunity for people on the receiving end of such knowledge production to relate it to what they knew from what happened to them and each other. The result was their highlighting of the dissonance between their portrait of a messy, confused, often ineffectual and abusive system, compared to one that had predominantly only been subjected to the self-reinforcing analysis of the psych system and its allied researchers. History was effectively repeated and the view of disabled people when confronted with the conclusions of the Tavistock centre research about them that they would always be ‘social parasites’ [12, 32], was similarly echoed by the rising belief among survivors that such psych research was itself essentially unhelpful, inaccurate and parasitical. Survivor research was both a product and an important expression of the mental health service user/survivor movement as it had been of the disabled people’s movement (Sweeney et al., op. cit.).

Because of this, while lived experience has often come in for adverse criticism as only offering individual perspectives on direct experience, it is also possible to develop more collective understandings. This is because such movements are essentially based on collective working, with participants having the opportunity to explore their experience with others who share it and as a result both to reassess themselves and that experience and also engage in a collective process enabling them to aggregate and synthesise such direct experience [32].

The increasing ambition to be more inclusive in such collective action also means that over time activist survivors both in their joint working and their survivor research have become increasingly aware of the importance of gaining a full picture by challenging prevailing exclusions and barriers, through their own ways of working. This is in contrast to conventional research, where for obvious reasons overlap between researchers and survivors has been limited and even where efforts have been made to address diversity with equality in sampling and other aspects of research, collation, analysis and interpretation of findings has remained with the researchers who tend to reflect such exclusions in their own make-up.

While data may be collated, even where group processes are used, the process is essentially an individual and individualizing one, shaped and determined by the researcher. With survivor research, participants no longer have to be reduced to telling their own story. The experience is collective – the obverse of conventional psy research processes and control can remain with survivors rather than being routinely appropriated by professionals. However, where there is an ambition for collaborative research with traditional researchers, then it is important for those in both roles to go through a process of unlearning and relearning to challenge these restrictions associated with conventional research.

### Ongoing challenges facing us

A key problem facing survivors and their movement in trying to challenge the psy system is that it talks the same language as they do. This applies both specifically in relation to the terminology associated with participation and involvement, where the two camps, as we have seen, use the same words to mean very different things. But it has also applied much more generally with many survivors still internalizing and operating within the terminology of ‘mental illness’ and ‘mental health’, however discordant it might be from their own experience and understandings. This is both to avoid being ruled out of prevailing discourse and to make sense to the many other survivors who are only familiar with this vernacular. That is one reason why the development of Mad Studies has been particularly helpful, since it explicitly rejects such terminology [44]). This opens up clear space between the two discourses and helps avoid the many problems arising from seeing them as essentially the same.

Madness and distress have long been associated with unpredictability, irrationality and violence. The crazed killer is an internationally established symbol of threat, horror and nightmare. While this association seems timeless, it was strongly resuscitated in the late twentieth century by the introduction in western societies of ‘care in the community’ as the large nineteenth century institutions were coming to the end of their physical life and policy acceptability. The introduction of ‘community care’, usually on the cheap without adequate resourcing or provision, resulted in the re-association of mental health service users with danger and violence. While there was little if any evidence to justify this new coupling, it certainly exacerbated policy and public fear and hostility against mental health service users, encouraged both their neglect and the emergence of hate crime. However, as well as highlighting that emerging problems were at least as much about the inadequacy of psych services, it also focused attention again on the violent aspects of psych services, particularly in relation to some of the most vulnerable service users; Black and minoritized people, indigenous people and refugees. A new concept of ‘slow violence’ was developed to help us understand ways in which the psych system and associated public policy could restrict service users’ rights and even remove their rights, without adequate protection [45].

As we have seen, one of the many characteristics that the survivor movement has in common with other NSMs is the emphasis it places on the specific experience and identity of those involved as a basis for reinterpreting their identities and experience together. This does not mean that mental health service users necessarily see themselves as a homogeneous, discrete and separate group. We also stress our overlaps and intersectionality. We highlight the difference due to our lived experience and specific oppression as a basis for campaigning – campaigning as a basis for our recognition for who we are and to be included as such. Thus, we are calling for inclusion, not integration, acceptance, nor assimilation. Similarly, when we argue that madness and distress are not symptomatic of some particular disordered individual or group, we are not making some kind of relativist statement that ‘we are all service users now’, or we all have ‘mental elf’, etc. This has emerged as part of professional PPI discussions, as if the possibility that at some point someone may be subjected to such experience entitles them to comment as an expert now. This is clearly not the case, whether the issue under discussion is sexual assault, disability or being a trans person. Similarly suffering some emotional hurt or moments of psychological difficulty is not the same as getting sucked long term into the psychiatric system or being labelled as ‘schizophrenic’. Clearly there may not be agreement about where lines should be drawn and equally the psych system’s increasing propensity to have a diagnosis for almost everything confuses the issue even further.

What we are saying is that madness and distress are something that can happen to and affect anyone (although many social factors determine which groups *are* affected) but unless and until they and the psy responses have happened to you, you are not in position to pontificate on the subject or speak for those who do have such direct experience. Similarly suggesting that we should all be talking more about our ‘mental health’ ignores the negatives and hostilities this may incur because of the hardening of negative attitudes towards mental health service uses/survivors stoked up by government and government departments, for example, as part of its punitive and exclusionary approach to disability welfare benefits.

The positivist values that psychiatric and indeed much health research are built on are traced to the eighteenth century enlightenment and its commitment to ‘science’, measurement and experiment. The principles of neutrality, objectivity and distance were subsequently extended from the natural sciences to social and health sciences where they became a western gospel for achieving rigour, reliability and replicability in research. This history helps explain many conventional objections to user involvement and the subjectivity associated with such research. However, such objections take no account of the way in which the natural sciences have themselves changed with the move from Newtonian to recognition of relativity and quantum theory [46]. What these highlight are the potential limits of Newtonian physics and the relation and interaction between observation and phenomenon.

### Principles for understanding and action

The evidence suggests that many mental health service users when asked feel that the medicalised individual model of mental illness is inadequate as a basis for making sense of and responding to their situation [47, 48]. A number of clear principles have emerged amongst survivors and their organisations internationally for challenging traditional understandings of their experience. These include being:

- treated as an equal human being with full citizen rights
- able to speak and act for yourself
- able to access the support and help that you find helpful
- protected from ‘treatment’ based on restriction of your rights
- equal opportunities with non-service users

and so on. While these amount to a clear programme for involvement and empowerment, they do not represent a distinct philosophy in the same way as the social model of disability developed by the disabled people’s movement. Here we are not suggesting that the dominant medicalised understanding of madness and distress needs to be replaced by another monolithic belief system. This would be at odds with efforts to decolonize madness and support survivors to develop our own culturally and politically sensitive and grounded analyses and responses to our situation. But what we do believe here is that such philosophical underpinnings, in all their variety, consistent with ethnic and cultural difference, aspirations to decolonize and to support the rights of indigenous people’s, are likely to be a strong precondition for challenging prevailing psy mental illness models and resisting their continuing imposition [49]**.**

### Is there another opening up?

For all that conventional research and thinking have adopted PPI and made it their own and for all that many, many more activities are dubbed with the initials ‘PPI’, there are signs that the institutional failure of this endeavour is not complete. Or rather, that work continues and flourishes outside its perimeter. For instance, the EURIKHA project which interviewed 58 people in the Global North whom we referred to as ‘knowledge-makers and activists’ found evidence that many strands of positive work was taking place. Interestingly, the more marginalised the group doing this work, the more radical their arguments and actions tended to be. Perhaps this was because they had nothing to lose because being white and middle class was associated with a position (usually very precarious) in academia and so there was pressure to conform. Those outside this space had less constraints on breaking boundaries – actual and conceptual – and so they were less of a threat to the mainstream simply through absence. The work they were doing also threw into question the extent to which mental health was a matter of ‘health’ One does not need to go down the Szazsian route to make that argument. To propose that what we call ‘mental health’ is a socially structured matter of daily living does of course fly in the face of one of psychiatry’s fundamental assumptions—that madness is a medical matter [50]. This assumption was not questioned in involvement spaces at all. But it was questioned elsewhere and otherwise, hidden for the most part but bubbling under the surface. And with very practical implications for support especially in times of crisis when authorities are most likely to step in.

One could argue also that the mainstream is imploding, by which we mean that it is not living up to its own standards. Although it has been interrogated remorselessly, the ‘Recovery’ literature has ceased to be a coherent body of work, we would argue. The lead term itself – ‘Recovery’ – now seems to have as many meanings as there are people who write about it [51]. Critiques are put – for instance that the approach is normalising – the critique is denied and things go on as before. [52] But the range of empirical studies is now enormous although it is accompanied by a poverty of theory. That is just an example and applies to many central ideas in this field. More formally, a ‘crisis of replication’ has been noted in the field of psychology. [53] A study may appear to show that a procedure is effective but it is found to be impossible to replicate that result. This appears endemic and if one of the assumptions is that knowledge building proceeds by the gradual accumulation of empirical facts, the discipline is in real trouble of those ‘facts’ are so evanescent and slippery.

Some prominent figures in the field have started to take this state of affairs seriously. In respect of psychopharmacology, Rose [15] quotes many leading researchers, amongst them Thomas Insel, who now believe that no new developments are on the table. “The pipeline is empty.” Here is a fairly lengthy quote from Insel:

I spent 13 years at NIMH really pushing on the neuroscience and genetics of mental disorders, and when I look back on that I realize that while I think I succeeded at getting lots of really cool papers published by cool scientists at fairly large costs–-I think $20 billion–-I don’t think we moved the needle in reducing suicide, reducing hospitalizations, improving recovery for the tens of millions of people who have mental illness,” Insel says. “I hold myself accountable for that.” [54]

Most pharmaceutical companies have withdrawn from the area of CNS drug development apart from those aimed at the Alzheimer group of conditions. Of course, a drop in profits was the major driver, but some did have the courage to say that nobody had been helped by the output of this major industry. Again, Robin Murray, one of the most highly-cited psychiatric researchers in the world, has written two papers critically reflecting on his life’s work in biomedicine. One is called “Mistakes I have made in my psychiatric career” and the other argues that maintenance medication is perhaps not such a good idea after all [55, 56]. Recently, Murray gave a talk at the annual Royal College of Psychiatrists conference in 2023 addressing how psychiatry should respond to its critics. He featured 5 film clips of these ‘critics’, two of them survivors. His ‘approach’ is not aggressive but ‘collaborative’ (his terminology) but he is moving the dial away from genetic to social determinants of mental distress.. And just this year Vikram Patel and colleagues published a paper that argued “Business as usual has failed. Business as usual will continue to fail” [57]. Addressing mental health globally, the paper is not quite as radical as these ‘facts’ would imply. But once again, Vikram Patel is an extremely influential figure.

Finally, a group of survivors gave a paper at the 2023 Annual Conference on Schizophrenia. Their presentation was on participatory research. This is an extremely conservative organisation but the paper drew one of the largest audiences at the whole conference. Participatory research, as we remarked, does not even feature in the ‘hierarchy of evidence’ so the presence of venerable psychiatrists at such a presentation suggests they are checking out survivor research and perhaps becoming disenchanted with the hierarchy itself and the assumptions it embodies.

## Conclusion

What then does all this have to do with PPI, Patient Involvement? We have argued that such involvement failed because it could not or would not break the fundamental assumptions that underpin psychiatric research. The point of the above remarks is to suggest that, helped along by the efforts of some survivors, these assumptions are becoming fragile. Some will say that Murray’s ‘mea culpa’ is having your cake and eating it. Only a person in such a prominent position could do this without risk to his reputation at least. Alternatively, only such a prominent figure could make such public statements and have them listened to. It is a sad fact that the user movement has been making these points for decades but they have generally been dismissed as the utterances of complaining patients. It becomes a different matter if the Thomas Insels and Robin Murrays of this world make them. Then they begin to break normal science which, as Kuhn observed, is at a moment replete with danger. So maybe there is a new space for a different kind of patient involvement where these assumptions are both surfaced and questioned rather than lie both fallow and dormant.

Should this undoing occur, what would take its place? Many things no doubt, but since this piece is about research we should broach the question of what it is conceptually that might underpin Mad Knowledge. ‘Underpin’ is deliberately ill-defined because, in our view, the days of general epistemologies are over so at least we are thinking in plurals not singular generalities and also not in rigid classifications. One such is the ‘rational subject’ – that which we are not. So do we want to say we *are* rational subjects? Not in the Enlightenment sense of the isolated cognitive individual. We have said enough about this to show that it is a historically and geographically specific idea and other societies conceptualise the ‘self’ differently – as malleable, interconnected, exposed to adversities of multiple kinds as well of course as exposed to facilitative surroundings. We are already beginning to see expressions of this in Global South countries, sometimes drawing on western thinking, like the UN Convention on the Rights of People with Disabilities and sometimes drawing on indigenous concepts like Ubuntu [49].

We do not want to normalise but to increase the bandwidth of what it means to be human. Psychiatry, we have both argued in different places, has no theory of the social. Indeed we have gone further and said the survivor movement too is lacking in this respect. [47, 58]. Rejecting general epistemologies is not to resist foundational concepts. We need to replace and reconfigure psychiatry’s decontextualised individual. We need concepts like structural violence to understand the harms systems do to groups and individuals. AND we need to understand the barriers to making these changes and much **more**. PPI may have brought issues of involvement and empowerment into the mainstream. Survivor research and specifically Mad Studies are redefining their meanings and possibilities in the context of psychiatry.

## Data Availability

All data generated or analysed during this study are included in this published article [and its supplementary information files].
